# Development of Metal-Enhanced Fluorescence Nanorods on Micro Post Arrays for Portable Detection of Human Semen Biomarkers

**DOI:** 10.3390/mi16121378

**Published:** 2025-12-02

**Authors:** Seongmin Lee, Won Il Heo, Kui Young Park, Seong Jun Seo, Xun Lu, Seok-min Kim

**Affiliations:** 1 Department of Mechanical Engineering, Chung-Ang University, Seoul 06974, Republic of Korea; 2 Department of Dermatology, Chung-Ang University Hospital, Seoul 06973, Republic of Korea; 3 Department of Mechanical Engineering, Yanbian University, Yanji 133002, China; 4 Department of Computer Science and Engineering, Chung-Ang University, Seoul 06974, Republic of Korea

**Keywords:** micro array, glancing angle deposition, metal nanorods, UV imprinting process, portable device

## Abstract

Rapid and reliable on-site identification of body fluids is essential in forensic and field diagnostic applications. Commercial kits provide only single results and often suffer from cross-reactivity, while conventional microarrays offer multiplex capability but lack sufficient fluorescence intensity for field-deployable systems. In this study, we present a highly sensitive nanorods on micro post array (NMPA) substrate and a smartphone-based portable detection system. The NMPA substrate integrates metal nanorods with UV-imprinted micro post structures to produce metal-enhanced fluorescence and improved signal localization. When evaluated using a microarray scanner, the substrate achieved high sensitivity, detecting semen diluted up to 1/100,000. The portable smartphone system further demonstrated simultaneous detection of three semen biomarkers (PSA, ACPP, and Semenogelin-1) at a 1/1000 dilution, matching the detection limit of commercial kits. Specificity tests using blood, saliva, urine, vaginal fluid, and environmental contaminants showed no false-positive responses. These results highlight the potential of the NMPA system as a portable diagnostic technology capable of rapid (<15 min), multiplex, and highly sensitive detection in field environments. Future work will focus on quantitative calibration, substrate stability assessment, and expansion toward multi biomarker panels for broader forensic and clinical applications.

## 1. Introduction

Biological evidence, such as blood, semen, and saliva, plays a critical role in forensic investigations, even though these samples are often collected in small quantities, originate from diverse environments, and may exist as complex mixtures [1]. Although blood, semen, and saliva are the most encountered forms of liquid biological evidence, other bodily fluids such as vaginal fluid, urine, and sweat may also be present [2]. Because of these complexities, rapid and accurate on-site identification of biological fluids is crucial for ensuring reliable downstream analysis and preventing the loss or contamination of valuable samples [3]. In current forensic practice, multiple commercial detection kits are frequently used in parallel to increase analytical accuracy [4]. However, the single result limitation of these tests may lead to inefficient utilization or loss of critical samples. Therefore, there is a growing demand for on-site analytical platforms capable of extracting maximal information from minimal sample volumes.

Microarray technology immobilizes capture probes or antibodies onto a microscale area, enabling simultaneous detection of multiple protein biomarkers using only a small sample volume [5]. However, because fluorescent signals are generated within confined microscale regions, the resulting intensity is inherently weak [6]. Consequently, conventional microarray platforms require highly sensitive and specialized fluorescence scanners, which limit their practicality for on-site or portable applications. Overcoming the intrinsically low signal intensity of microarrays therefore remains a critical challenge for integrating such platforms into portable detection systems.

Metal-enhanced fluorescence (MEF) technology has garnered attention as an effective solution to overcome the weak signal intensity in microarray systems [7]. MEF occurs when fluorophores are positioned near metallic nanostructures that support localized surface plasmon resonances (LSPR), resulting in enhanced excitation fields, increased radiative decay rates, and improved quantum yield. Among various plasmonic architectures, nanogap-induced hotspots (typically 10–30 nm) are known to generate particularly strong electromagnetic field enhancement [8]. In addition, Ag nanostructures with diameters of approximately 100 nm have also been reported to enhance fluorescence within the 500–700 nm wavelength range, which is widely used in bioassays [9]. These characteristics allow MEF substrates to substantially improve the sensitivity and reliability of fluorescence-based detection, even at low analyte concentrations [10].

Despite these advantages, large-area fabrication of metallic nanostructures for practical microarray biosensing remains challenging. Techniques such as electron-beam lithography offer precise nanoscale control but are expensive, slow, and impractical for producing large-area substrates (25 × 75 mm^2^). Alternative approaches, including wet-chemical synthesis and electrochemical deposition, often suffer from poor reproducibility and limited structural control [11]. Therefore, a fabrication strategy that is cost-effective, scalable, and capable of producing nanostructures over large substrates is essential for developing MEF platforms suitable for clinical and biotechnological applications.

The glancing angle deposition (GLAD) technique provides a promising solution to these challenges. GLAD is a physical vapor deposition method that creates metallic nanostructures by placing the substrate at a high oblique angle relative to the incoming vapor flux. Initial nano-islands cast shadowed regions that promote the growth of isolated nanostructures, and continuous substrate rotation produces vertically oriented, columnar nanorods [12]. The technique is low-cost, highly scalable, and compatible with large substrates, making it increasingly attractive for producing MEF platforms for biomolecular detection [5]. In our previous work, Ag nanorods fabricated on a micro post array demonstrated more than a 10-fold increase in fluorescence intensity compared with amine-coated glass slides and epoxy-coated microarray substrates [13].

In this study, we applied a metal nanorod on a micro post array (NMPA) structure to overcome the inherent limitations of conventional micro arrays and to enable integration into portable detection systems. In addition, to facilitate practical on-site applicability, we reduced the overall detection process to 15 min and demonstrated fluorescence imaging using a smartphone-based system. These results confirm the feasibility of the platform for portable, point-of-care applications.

## 2. Materials and Methods

### 2.1. Fabrication of Metal Nanorods on Micro Post Arrays (NMPAs)

In this study, a micro post array with a 300 μm diameter, 50 μm height, 600 μm pitch, and a patterned area of 75 × 25 cm^2^ was introduced to fix the position of detection spots. The micro posts, with their height differences, serve to immobilize the shape and location of the bioreceptor spots on the top of the posts. This design emphasizes the signals from the top of the micro posts, where focusing is aligned, while shifting the noise signals from the surrounding area. This effectively improves the signal-to-noise ratio, ensuring higher detection reliability [13].

**Figure 1:**
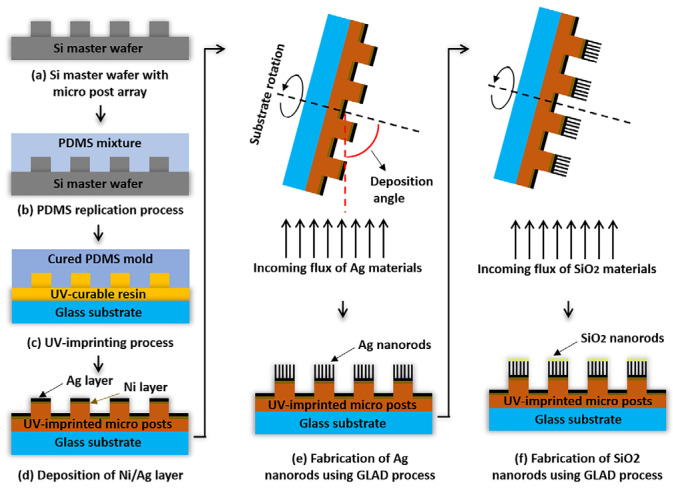
Schematic illustration of the fabrication process of the metal nanorod on the micro post array (NMPA) substrate. (**a**–**c**) UV micro imprinting process used to replicate micro post structures on a glass substrate using a PDMS mold. (**d**) Deposition of 10 nm Ni and 50 nm Ag layers to enhance surface adhesion prior to GLAD. (**e**) Ag nanorods are deposited onto the micro posts using glancing-angle deposition (GLAD). (**f**) A subsequent SiO_2_ GLAD step forms smaller SiO_2_ nanorods on top of the Ag nanorods, improving the adhesion of biomolecules to the substrate.

The micro post structures were fabricated on a glass substrate using a UV-imprinting process. Subsequently, before the GLAD step, a 10 nm nickel layer and a 50 nm silver layer were deposited onto the substrate to enhance adhesion. Metal nanorods were fabricated on micro posts using the GLAD process. Ag nanorods with a height of 300 nm were deposited by setting the angle between the deposition direction and the substrate to 85° and the substrate rotation speed to 5 rpm. To further enhance protein immobilization capabilities, an additional 15 nm of silicon dioxide was deposited. The overall schematic can be seen in Figure 1.

**Figure 2:**
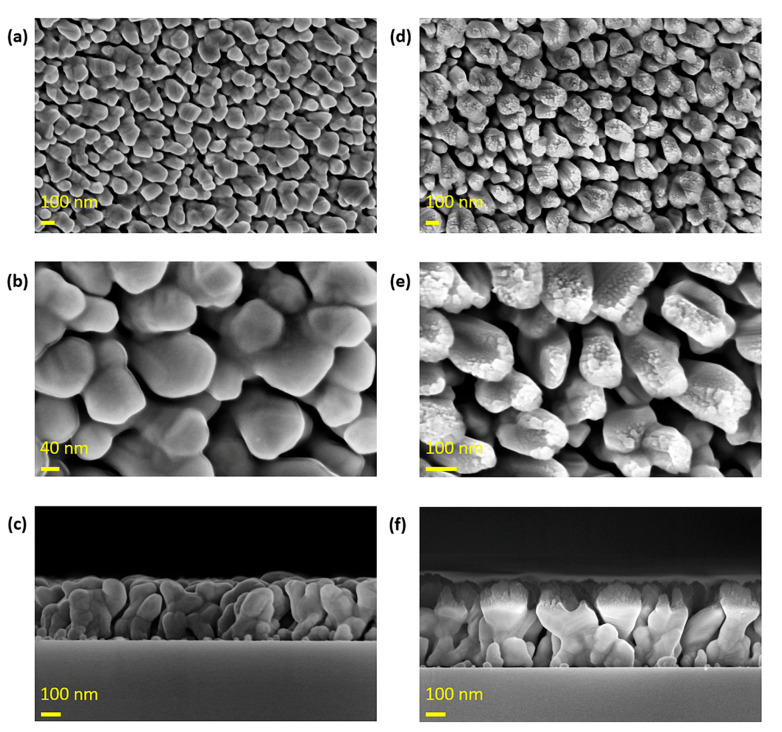
SEM images of nanorods fabricated using the GLAD process. (**a**–**c**) Ag nanorods deposited on the micro post surface, showing (**a**) top-view morphology, (**b**) magnified view of Ag nanostructures, and (**c**) cross-sectional view revealing their growth. (**d**–**f**) SiO_2_ nanorods formed on top of the Ag nanorods through a subsequent GLAD step, shown as (**d**) top-view, (**e**) magnified surface morphology, and (**f**) cross-sectional structure.

The morphology of the fabricated metal nanorods was characterized using scanning electron microscopy (SEM). As shown in Figure 2, Ag nanorods with an approximate diameter of 100 nm were successfully formed, and a uniformly deposited ultrafine SiO_2_ nanorod layer was observed on their top surfaces. It was confirmed that the additionally deposited SiO_2_ was deposited only on the top surface of the existing Ag nanorods. This indicates that the additional SiO_2_ nanorod structure does not affect the Ag nanogaps responsible for fluorescence enhancement. The ultrafine SiO_2_ nanorod provides hydroxyl (-OH) sites during the subsequent coating process, which is carried out to enhance the adhesion between the antibody and the substrate surface.

### 2.2. Selection of Semen Biomarkers and Capture Antibodies

The selection of appropriate biomarkers is essential for developing an effective multiplex detection system. Biomarkers must exhibit high specificity and sensitivity to the target protein while remaining stable under diverse environmental conditions. Additionally, compatibility with the detection platform, such as fluorescence-based microarray systems, is essential to ensure accurate and reliable results. In this study, biomarkers were carefully selected to enable the simultaneous detection of multiple analytes, addressing the diverse needs of on-site analysis and enhancing the system’s overall utility.

For multiplex microarray detection, biomarkers with high specificity to semen were selected: prostate-specific antigen (PSA), prostatic acid phosphatase (ACPP), and Semenogelin-1. These biomarkers are routinely used in commercial semen detection kits, including PSA immunochromatographic kits, SM reagent ACPP colorimetric assays, and RSID immunochromatographic tests [14,15,16].

The selection of an appropriate capture antibody is a critical step in microarray-based biomarker detection. Although multiple commercial antibodies may target the same protein, their performance can vary substantially due to differences in affinity, epitope accessibility, and surface-immobilization behavior. Interactions between the antibody and the metal nanostructure can influence binding orientation and effective probe density, ultimately affecting the fluorescence signal intensity. Therefore, evaluating and comparing candidate capture antibodies is essential to ensure reliable and sensitive detection on the NMPA substrate.

In this study, we systematically evaluated three PSA capture antibodies, two ACPP capture antibodies, and two Semenogelin-1 capture antibodies to identify the most suitable candidates for Ag nanorods microarray detection. To ensure a consistent comparison, a Ag nanorod substrate was prepared by depositing 10 nm of Ni and 50 nm of Ag onto a glass slide, followed by the fabrication of 300 nm Ag nanorods using the GLAD process. Each capture antibody solution was spotted in seven replicates at 1 mm intervals with a dispensing volume of 15 nL per spot, and the substrate was incubated at 4 °C for 15 h to facilitate antibody immobilization.

**Figure 3:**
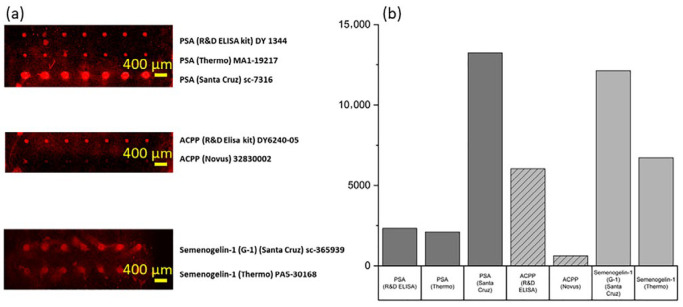
Evaluation of capture antibodies for PSA, ACPP, and Semenogelin-1. (**a**) Fluorescence microarray images for candidate antibodies. (**b**) Quantitative fluorescence comparison used to identify optimal antibodies.

The performance of each antibody was assessed following the respective experimental protocols, and the results are presented in Figure 3. A comprehensive summary of the final selected capture antibody is provided in Table 1.

### 2.3. Fabrication and Functionalization of the NMPA Biochip

Metal-based substrates are generally less stable than DNA microarray platforms and can present challenges in the fabrication of protein chips. In particular, the multiple liquid-handling steps involved in protein microarray processing, such as blocking, incubation, and washing, can cause significant protein loss if the biomolecules are not firmly immobilized on the surface. This results in decreased signal intensity and reduced accuracy, posing significant challenges to the reliability of protein chip performance.

**Figure 4:**
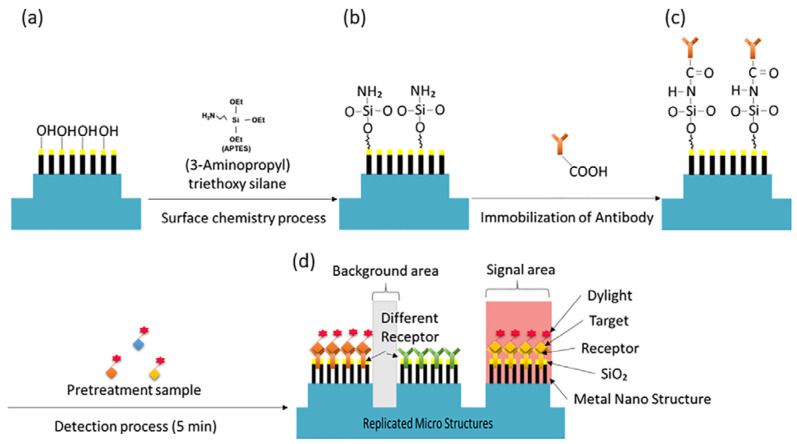
Schematic of NMPA surface functionalization and detection workflow. (**a**) SiO_2_ nanorod surface. (**b**) APTES functionalization. (**c**) Capture antibody immobilization. (**d**) Conceptual diagram of the NMPA structure after interaction with the sample.

To further enhance the protein immobilization capability on the surface, even after SiO_2_ deposition, 3-Aminopropyl triethoxy silane (APTES, Sigma Aldrich, Saint Louis, MO, USA) was vapor deposited at 90 °C for 30 min to modify the surface properties of the metal substrate [17]. Subsequently, capture antibodies for the selected biomarkers (PSA (250 μg/mL), ACPP (250 μg/mL), and Semenogelin-1 (250 μg/mL)) were diluted in 1 × PBS and dispensed onto the top of each micro post at a volume of 15 nL per spot. The substrate was incubated overnight at 4 °C for 15 h. Finally, washing and blocking steps were carried out using washing buffers (0.05% Tween 20 in PBS) and blocking buffers (R&D Systems, Minneapolis, MN, USA) to prevent non-specific binding. These procedures completed the preparation of the NMPA biochip for subsequent fluorescence-based detection, as illustrated schematically in Figure 4.

For fluorescence-based detection, samples were fluorescently labeled using the Dylight 633 Fast Conjugation Kit (Abcam, Cambridge, UK). Sample pretreatment consisted of mixing 10 μL sample with 1 μL Modifier reagent and 0.25 μL Dylight 633 dye (incubation: 5 min), followed by the addition of 1 μL Quencher solution (incubation: 4 min). The labeled sample was applied to the NMPA and incubated for 5 min. After a single DI water rinse, fluorescence signals were measured immediately.

### 2.4. Design of the Portable Detection System

**Figure 5:**
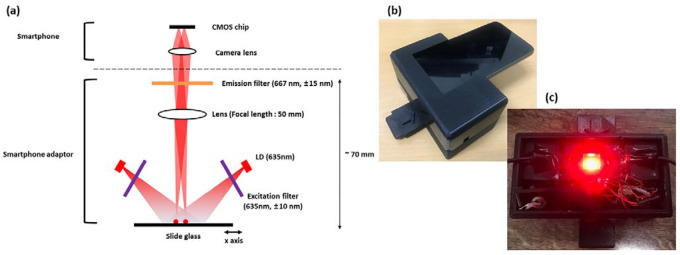
Portable smartphone-based fluorescence detection system. (**a**) System schematic showing laser diode, optical filters, and lens. (**b**) Photograph of assembled system. (**c**) Internal view of optical configuration in the smartphone adaptor.

A portable detection system was developed to utilize the metal NMPA for on-site applications (Figure 5). The system targeted the fluorescent dye Dylight 633, with an excitation wavelength of 638 nm and an emission wavelength of 658 nm. A laser diode with a center wavelength of 635 nm and a power output of 20 mW was employed as the excitation source. The excitation and emission filters (CHROMA Technology Corp., Bellows Falls, VT, USA) were designed with center wavelengths of 635 nm and 667 nm, respectively, and bandwidths of 10 nm and 15 nm.

The emitted light from the laser diode passed through the excitation filter, allowing only wavelengths shorter than 645 nm to transmit. This filtered light interacted with the fluorescent dye, generating fluorescence with a center wavelength of 658 nm. The fluorescence then passed through the emission filter, permitting only wavelengths longer than 652 nm to reach the portable device. Additionally, a lens with a focal length of 50 mm was placed in the optical path to ensure that the image was focused on the portable device. This design enabled precise fluorescence detection and real-time data acquisition in a compact and field deployable system.

Fluorescence imaging was performed using an LG G6 smartphone operated in manual mode (ISO 3200 [18], 1/10 s exposure, fixed focus, no digital zoom). The adaptor ensured a fixed alignment geometry and constant sample-to-camera distance to minimize user variability. Image processing was performed using ImageJ (version 1.53t) and included background subtraction, selection of circular regions of interest (ROIs), and extraction of mean fluorescence intensity.

A full calibration workflow (reference slides, controlled exposure protocol) was not implemented in this feasibility study; this limitation will be addressed in future system development. A summary of the physical and optical specifications of the portable detection system is provided in Table 2.

**Table 2:** Specifications of the portable fluorescence detection device.

Category	Specification
Device dimensions	120 × 65 × 55 mm
Weight	180 g (excluding smartphone)
Excitation source	635 nm laser diode (20 mW)
Excitation filter	625–645 nm bandpass
Emission filter	652–682 nm long-pass
Collection optics	50 mm focal-length lens
Power supply	3.7 V Li-ion battery
Battery life	~4–5 h intermittent use

## 3. Results and Discussion

### 3.1. Performance Evaluation of the NMPA as a Microarray Platform

**Figure 6:**
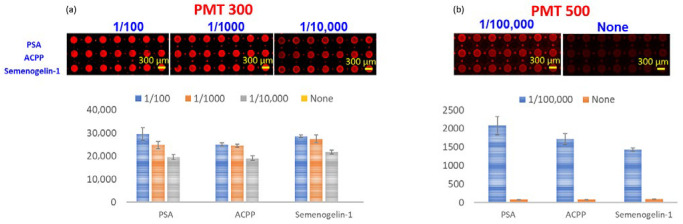
Fluorescence detection performance of the NMPA microarray substrate using a commercial scanner. Signals remain detectable up to a 1/100,000 dilution, demonstrating strong metal-enhanced fluorescence and high sensitivity.

The fluorescence performance of the NMPA substrate was first evaluated to determine its capability as a highly sensitive microarray platform (Figure 6). Semen samples were serially diluted in 10-fold increments, and fluorescence signals were measured using a microarray scanner (GenePix 4000B, Molecular Devices, San Jose, CA, USA).

At a photomultiplier tube (PMT) gain of 500, the strong metal-enhanced fluorescence generated by the NMPA resulted in signal saturation for semen dilutions up to 1/1000, demonstrating the significant amplification effect of the Ag nanorod–micro post architecture. To enable quantitative measurements at higher dilutions, subsequent scans were performed at a reduced PMT gain of 300.

Under these scanning conditions, the NMPA microarray demonstrated detectable fluorescence signals at dilutions as high as 1/100,000. These results confirm that the NMPA exhibits high sensitivity microarray performance, supporting its potential use in applications requiring high signal amplification and low detection limits.

### 3.2. Evaluation of Sensitivity and Specificity

To assess whether the NMPA can reliably differentiate semen from other biologically or environmentally relevant fluids, sensitivity and specificity tests were conducted using semen, male urine, female urine, vaginal fluid, blood, saliva, coffee, and detergent. The results were compared with those obtained from commercial PSA kits, SM ACPP tests, and RSID semen kits.

Commercial PSA kits exhibited clear cross-reactivity, producing positive bands for male urine despite the absence of semen. Similarly, the SM reagent test displayed nonspecific color development in response to urine and several other interfering fluids. These results highlight the susceptibility of conventional rapid tests to false positives when complex mixtures are present.

**Figure 7:**
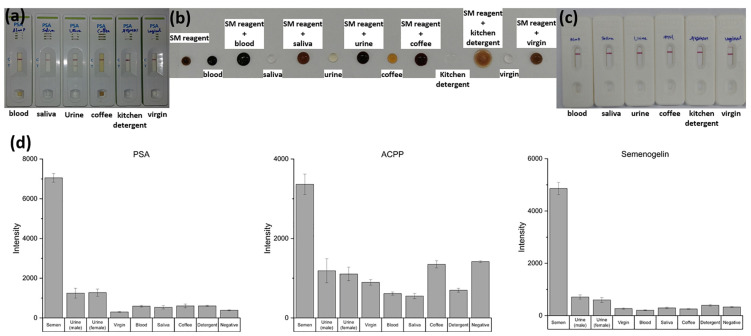
Evaluation of sensitivity and specificity across commercial tests and the NMPA substrate. (**a**) PSA kit, (**b**) SM reagent, (**c**) RSID kit, (**d**) NMPA substrate (For the NMPA substrate, the results are shown (from left to right) for semen, urine (male and female), vaginal fluid, blood, saliva, coffee, detergent, and the negative control). The NMPA platform produced strong fluorescence signals only for semen, with minimal background from all other matrices, confirming high specificity and no false-positive responses.

In contrast, the enhanced fluorescence NMPA substrate demonstrated strong specificity for semen biomarkers (PSA, ACPP, and Semenogelin-1). As shown in Figure 7d, semen samples produced significantly higher fluorescence signals than all other tested matrices, while blood, saliva, urine, vaginal fluid, coffee, and detergent produced minimal background signals comparable to the negative control. No false-positive signals were observed across any non-semen fluids.

These findings demonstrate that the NMPA provides superior specificity compared with existing commercial kits and is well suited for on-site forensic applications where accurate discrimination among complex biofluids is essential.

### 3.3. Evaluation of Field Applicability Using the Portable System

**Figure 8:**
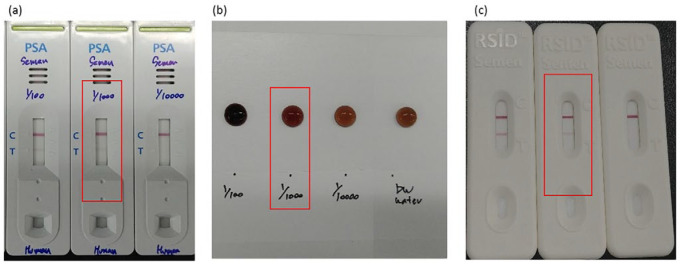
Detection limit comparison using commercial test kits. (**a**) PSA rapid test kit, (**b**) SM ACPP colorimetric test, (**c**) RSID semen kit. All exhibited detection limits of approximately 1/1000 semen dilution.

To evaluate whether the developed NMPA biochip and portable imaging system are suitable for real-time, on-site applications, semen detection performance was compared with the detection limits of commercial kits. As shown in Figure 8a–c, commercially available PSA kits, SM reagent tests, and RSID semen kits consistently detected semen only up to a 1/1000 dilution. This dilution level therefore represents the practical on-site detection limit for current forensic field-use devices.

**Figure 9:**
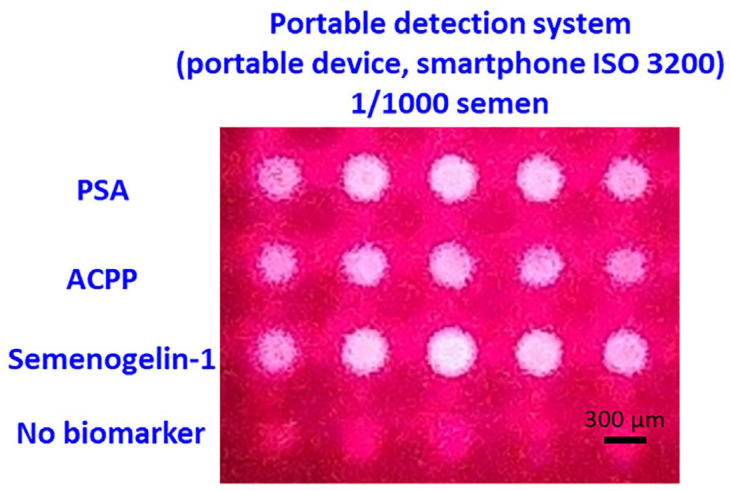
Smartphone-based fluorescence detection of 1/1000 diluted semen on the NMPA substrate. Strong fluorescence signals were observed exclusively at biomarker capture spots.

Using this dilution benchmark, the developed portable system was tested with NMPA substrates using semen samples diluted 1/1000. Clear and well-defined fluorescence signals were observed for PSA, ACPP, and Semenogelin-1 capture spots, while the antibody-free control row showed only minimal signals. These results confirm that the portable NMPA detection system can reliably detect semen at the same limit as commercial kits, while additionally providing multiplex detection capability (Figure 9).

Fluorescence images were acquired using an LG G6 smartphone operating in manual mode (ISO 3200, 1/10 s exposure, fixed focus, no digital zoom). Only background subtraction was applied prior to fluorescence quantification. The total detection time (including sample conjugation, incubation, washing, and imaging) was under 15 min, matching the operation time of commercial kits but providing significantly more analytical information.

### 3.4. Limitations of the Current Portable Detection Study

The portable detection results reported here represent feasibility demonstrations rather than full statistical validation. Multi-sample and multi-run replicates were not performed; therefore, quantitative metrics such as LOD, standard deviation, and reproducibility could not be established. These limitations will be addressed in future studies through systematic calibration procedures and repeated measurements across independent samples.

Additionally, long-term substrate stability (including storage shelf-life, resistance to humidity and temperature fluctuations, and fluorescence degradation under light exposure) was not evaluated. These characteristics are essential for commercial translation, and accelerated aging and environmental stress tests are planned for future work.

Regarding reusability, the NMPA chips are coated with a blocking buffer to suppress nonspecific binding prior to biomarker immobilization. Once an assay is performed, the blocking layer is partially consumed. We observed increased nonspecific background signals after reuse, indicating a loss of blocking efficiency. For this reason, the present NMPA substrates are treated as single-use disposable chips.

### 3.5. Cost Estimation and Scalability

To estimate material cost, it was assumed that 40 glass slides are processed per GLAD run, producing 40 NMPA chips. Approximately 26 g of Ag was consumed per run (~0.65 g per chip), corresponding to USD ~1.0–1.2 of silver cost per chip based on current pricing (1.7 USD/g). Glass slides contributed USD ~0.6–1.4 each, and UV-curable resin added only a few cents, resulting in a substrate material cost of approximately USD 2.6 per chip at the laboratory scale.

Capture antibodies were spotted at 250 μg/mL and 15 nL per spot (~3.75 ng). At typical market prices (3–5 USD/μg), the cost per chip is USD ~0.2 for antibody reagents. Dylight 633 conjugation consumes ~0.25 μL per test, corresponding to USD ~1–2 in dye cost. Therefore, the combined material cost per assay (substrate + antibodies + dye) is estimated to be USD 3–5, with substantial reductions expected in bulk manufacturing.

## 4. Conclusions

In this study, we developed a nanorods on micro post array (NMPA) substrate and an accompanying smartphone-based portable fluorescence detection system designed to overcome the inherent limitations of existing field-use semen detection technologies. Conventional rapid kits can detect only a single biomarker per test, leading to inefficient sample utilization, while traditional microarray platforms (despite their multiplexing capability) suffer from insufficient fluorescence intensity for portable deployment.

The NMPA substrate addresses these limitations by integrating metal nanorods with micro post structures, thereby maximizing signal intensity and reducing background interference. As a result, the substrate achieved a detection sensitivity of up to 1/100,000 semen dilution when evaluated using a microarray scanner.

Furthermore, the smartphone-based portable system successfully detected the three semen biomarkers (PSA, ACPP, and Semenogelin-1) simultaneously at a 1/1000 dilution, matching the detection limit of commercial kits while enabling multiplex analysis. These results demonstrate the potential of the NMPA platform to incorporate additional biomarker panels, enabling the simultaneous detection of multiple body fluids such as semen, vaginal fluid, saliva, and blood within a single assay.

Overall, this work presents a promising foundation for next-generation portable diagnostic tools capable of delivering rapid, multiplex, and highly sensitive analyses in field environments. Future studies will focus on expanding the biomarker library, improving quantitative reliability, and evaluating long-term substrate stability to further advance the practical deployment of this technology.

## Figures and Tables

**Table 1:** Selection of semen identification biomarkers and capture antibody.

Preliminary Test Method	Type of Biomarker	Capture Ab	Catalog No.
PSA Kit (ASAN PHARM, Bucheon-si, Gyeonggi-do, Republic of Korea)	PSA (Prostate-specific antigen)	PSA (A67-B/E3) Ab	Santa Cruz sc-7316
SM reagent (FUJI FILM Wako Pure Chemical, Osaka, Japan)	ACPP (Prostatic Acid Phosphatase)	ACPP Ab	R&D system DY6240-05
RSID Kit (Independent Forensics, Lombard, IL, USA)	Semenogelin-1	Semenogelin-1 (G-1) Ab	Santa Cruz Sc-365939

## Data Availability

The data presented in this study are available on request from the corresponding author. The data are not publicly available due to privacy and ethical restrictions related to human sample handling.

## Abbreviations

The following abbreviations are used in this manuscript:

NMPA	Nanorods on micro post arrays
MEF	Metal-enhanced fluorescence
LSPR	Localized surface plasmon resonances
GLAD	Glancing angle deposition
SEM	Scanning electron microscopy
PSA	Prostate-specific antigen
ACPP	Acid phosphatase
PBS	Phosphate-buffered saline
ROI	Regions of interest
CMOS	Complementary metal-oxide-semiconductor
PMT	Photomultiplier tube
